# Multi-Factor Cost Function-Based Interference-Aware Clustering with Voronoi Cell Partitioning for Dense WSNs

**DOI:** 10.3390/s26020546

**Published:** 2026-01-13

**Authors:** Soundrarajan Sam Peter, Parimanam Jayarajan, Rajagopal Maheswar, Shanmugam Maheswaran

**Affiliations:** 1 Department of Artificial Intelligence and Data Science, Sri Eshwar College of Engineering, Coimbatore 641202, Tamil Nadu, India; 2 Department of Electronics and Communication Engineering, Sri Krishna College of Technology, Coimbatore 641042, Tamil Nadu, India; p.jayarajan@skct.edu.in; 3 Department of Electronics and Communication Engineering, KPR Institute of Engineering and Technology, Coimbatore 641407, Tamil Nadu, India; maheswar.r@kpriet.ac.in; 4 Department of Electronics and Communication Engineering, Kongu Engineering College, Erode 638060, Tamil Nadu, India; mmaheswaran_eie@kongu.ac.in

**Keywords:** dynamic clustering, load balancing, density-aware clustering, node density, link quality

## Abstract

Efficient clustering and cluster head (CH) selection are the critical parameters of wireless sensor networks (WSNs) for their prolonged network lifetime. However, the performances of the traditional clustering algorithms like LEACH and HEED are not satisfactory when they are implemented on a dense WSN due to their unbalanced load distribution and high contention nature. In the traditional methods, the cluster heads are selected with respect to the residual energy criteria, and often create a circular cluster shape boundary with a uniform node distribution. This causes the cluster heads to become overloaded in the high-density regions and the unutilized cluster heads gather in the sparse regions. Therefore, frequent cluster head changes occur, which is not suitable for a real-time dynamic environment. In order to avoid these issues, this proposed work develops a density-aware adaptive clustering (DAAC) protocol for optimizing the CH selection and cluster formation in a dense wireless sensor network. The residual energy information, together with the local node density and link quality, is utilized as a single cluster head detection metric in this work. The local node density information assists the proposed work to estimate the sparse and dense area in the network that results in frequent cluster head congestion. DAAC is also included with a minimum inter-CH distance constraint for CH crowding, and a multi-factor cost function is used for making the clusters by inviting the nodes by their distance and an expected transmission energy. DAAC triggers re-clustering in a dynamic manner when it finds a response in the CH energy depletion or a significant change in the load density. Unlike the traditional circular cluster boundaries, DAAC utilizes dynamic Voronoi cells (VCs) for making an interference-aware coverage in the network. This makes dense WSNs operate efficiently, by providing a hierarchical extension, on making secondary CHs in an extremely dense scenario. The proposed model is implemented in MATLAB simulation, to determine and compare its efficiency over the traditional algorithms such as LEACH and HEED, which shows a satisfactory network lifetime improvement of 20.53% and 32.51%, an average increase in packet delivery ratio by 8.14% and 25.68%, and an enhancement in total throughput packet by 140.15% and 883.51%, respectively.

## 1. Introduction

### 1.1. Motivation

Wireless sensor networks (WSNs) have emerged as one of the unavoidable technologies for various ranges of applications, including environmental monitoring, health care, smart cities, and so on. In these applications, WSNs are implemented to collect and transmit information to a central base station. WSNs are widely used due to their scalability, real-time monitoring capability, flexible deployment, and low-cost implementation. However, WSNs also face several challenges, such as limited energy, low memory and processing capability, signal interference, and security threats. The advantages and drawbacks of WSNs in real-world applications have been clearly discussed in recent studies [1,2]. Therefore, the performances of the WSNs are heavily affected with respect to their resource constraints, like limited energy availability and processing capabilities [3,4]. Therefore, there is always a need for making an efficient energy management system for ensuring a sustainable lifetime and for the reliability of the entire network. Clustering-based routing is one of the promising methods that has been extensively utilized for its efficiency in balancing energy consumption with a reduced communication overhead [5,6]. Traditional clustering algorithms such as Low-Energy Adaptive Clustering Hierarchy (LEACH) and Hybrid Energy-Efficient Distributed (HEED) protocol, that identify cluster heads (CHs) based on randomized or energy estimations, are computationally simple, but the performances of these algorithms are not satisfactory when they are implemented on high-density WSN applications [7,8]. Especially, random cluster formation makes the network develop uneven node distribution across clusters, which leads to overloaded CHs in some regions and underutilized CHs collect in sparse areas. Here, dense and sparse regions are distinguished based on the local node density, which effectively reflects the number of neighbouring nodes within the communication range of each sensor [9,10].

### 1.2. Challenge

This uneven distribution makes the network to change CHs frequently with high contention, which results in rapid energy depletion to degrade the network performance and lifetime [11,12]. Hence, there is a requirement for a clustering approach that considers both node density and residual energy, for its estimation on implementing in a high-density network deployment. In order to address these issues, density-aware clustering algorithms were introduced in recent years by integrating spatial node distribution estimations on the CH selection process. This makes the approaches to identify the crowded network region and sparse region for reducing the risk for CH congestion and irregular load balancing. Though there are some density-aware algorithms, all of them work on a fixed and circular boundaries, that fail to simulate the irregular distribution of nodes in real-time applications. These kinds of limitations, where interference and link quality are non-uniform, significantly reduce the network performance, which make such existing algorithms unsuitable in real-time dynamic situations.

### 1.3. Contribution

In this context, a density-aware adaptive clustering with Voronoi-based size limited clustering (DAAC+VC) is proposed with the following contributions:

- Cluster heads are selected using a composite metric that combines residual energy and link quality, along with the active node density in the network which is estimated on each round.
- Unlike traditional circular clusters, Voronoi-based partitioning ensures the network to be interference-aware and have spatially adaptive coverage.
- Additionally, the proposed model incorporates minimum inter-CH distance constraints and hierarchical extension, to manage extremely dense area with a secondary CH.
- The work triggers re-clustering in observation from the energy depletion and load variation on the CHs and the network nodes.

The following Section 2, explores the research attainments of the existing methods, Section 3 presents the complete concept behind the proposed approach, and its performance evaluations performed in MATLAB simulation are included in Section 4.

## 2. Literature Survey

### 2.1. Metaheuristic and Learning-Based Clustering Approaches

A Particle Swarm Optimization (PSO)-based clustering technique was proposed to balance the network nodes by incorporating double-exponential adaptive inertia weight and local exploitation. Its performance was compared against the fuzzy-based clustering approaches and K-means PSO, and found satisfactory, with its network longevity superior by a margin of 20% and 10%, respectively. A whale optimization technique was proposed for cognitive radio sensor networks to address the issue of early cluster failure due to energy imbalance and unstable clustering in the traditional approaches. This methodology was verified in an NS-2 platform, the energy efficiency and cluster size distribution were optimized, and an outcome of 15% better throughput and 12% better overhead over the traditional approaches was achieved [13]. An energy-aware clustering method was developed with an ink drop spread operator for clustering in WSNs. This was developed to maximize network lifetime by preventing premature node failures through balancing energy consumption. The algorithm was implemented in a dynamic network space where clustering and cluster head selection happens on each round. The experimental work shows that the proposed work outperforms the traditional LEACH and PEGASIS by at least 17% in terms of residual energy and network lifetime. Additionally, the work seems to be better in terms of having more active nodes compared to the existing techniques, and it provided a better clustering quality [14]. An improved squirrel search algorithm was designed for optimal cluster head selection in WSNs. The algorithm was implemented in MATLAB 2023a with enhancement on adaptive population initialization, dynamic step size count, and local search for a better convergence. The experimental work indicated an outcome of 88% PDR (packet delivery ratio), with a lower energy usage of 210 mJ, with a reduced cluster formation and cluster head selection time [15]. An improved Q-learning-based artificial bee colony algorithm was designed for optimal CH selection in a sensor network, where it reduced the energy consumption on multi-hop routing through reliable data transmission. It enhanced the exploration and exploitation of CH selection fuzzy logic-based weight assignment, and its simulation result indicated a better outcome on energy consumption, with 0.253 units for 1200 rounds. This extended the network lifetime of the model over the traditional LEACH and HEED approaches [16].

### 2.2. Multi-Hop, Fuzzy, and Swarm Intelligence-Based Clustering

An intra-cluster multi-hop-based cluster head rotation method was introduced to reduce unwanted re-clustering of nodes and CH rotation to save residual energy on each node with a pre-defined threshold value. The protocol was found to be satisfied on network lifetime and its stability over the existing algorithms [17]. A multi-level clustering approach with a modernized pufferfish optimization algorithm was developed to overcome the issues of regular clustering algorithms on poor sensor deployment. The work was integrated with a multi-level K-means clustering approach for addressing the premature convergence of sensor nodes, and that resulted in a 62.5% improvement on network lifetime and 29% on its energy consumption [18]. A fuzzy C-means clustering approach was integrated with a best and worst fitness-based sailfish whale optimization approach on cluster head selection for making an efficient routing process on the sensor networks. The work considered intra- and inter-cluster distances with residual energy, and its experimental study showed a betterment on throughput and packet delivery ratio, along with 50% energy savings over the WOA model [19]. A spotted hyena optimization model was proposed to improve the battery life of sensor nodes through cooperative hunting strategies to optimize exploration and exploitation in clustering. The simulation result showed a better stability with first node failure at 1300 rounds and 4% lower deployment cost [20]. A transient search optimization algorithm was developed for clustering in WSNs to reduce energy depletion and extend network lifetime. The approach was implemented in NS-2, and was found to achieve a 56.37% better network lifetime over the existing models [21].

### 2.3. Energy-Efficient, Hybrid, and Deep Learning-Based Clustering Approaches

An energy-efficient mega-cluster-based routing protocol was developed to overcome the hotspot problem in the WSNs. The performance was evaluated in a centralized fixed clustering model, where the base station partitions the network, and mega-clusterheads are rotated for uniform load distribution. The simulation result indicated a better network lifetime of 34.5% and significantly reduced the dead node counts over the existing approaches [22]. A spider wasp optimizer-based multi-hop routing was suggested to integrate inter-cluster routing with CH selection, where a communication distance factor and central relay points were used for such estimations. The simulation results indicated an improved network lifespan of 32.7%, and that outperformed the traditional approaches [23]. The traditional LEACH approach was integrated with the Artificial Neural Networks for making a faster response system for real-time applications. The approach was tested in MATLAB 2023a, where a dataset was generated for ANN training, and that provided an accuracy of 85% on the classification of CH, which was found to be faster than the traditional LEACH approach by 83.28% [24]. A metaheuristic-based dual cluster head selection with routing protocol was designed to address the non-deterministic polynomial-time problem on estimating CHs. A two-stage clustering process using a cheetah optimization algorithm and flower pollination algorithm was proposed to identify tentative CHs and final CHs on the network area. The work was also integrated with a carnivorous plant algorithm for an optimal route selection. The simulation result indicated a prolonged network lifetime of 1270 rounds, with a higher residual node energy of 24.48 at round 1000 [25]. A multi-objective-based clustering and routing approach using a tree hierarchical deep convolutional neural network with hybrid capuchin search and woodpecker mating algorithm was developed to improve the performances of WSNs, by avoiding rapid energy depletion and higher traffic near the base station. The approach was implemented in NS-2 simulation, where a significant performance improvement was achieved on all parameters, where 50% more alive nodes were observed in comparison with the existing methods [26]. Table 1 summarizes the attainments and limitations of the existing clustering techniques.

### 2.4. Research Gap

Despite several attainments observed from the existing methods, there are several research issues identified and categorized based on their common limitations:

- Optimization-based algorithms are noted with a premature convergence and local optima trapping while making the cluster and CH selection process, where it limits their adaptability in a high dynamic network condition.
- The metaheuristic algorithms were found to be computationally intensive, and that may not perform well when the number of nodes increases, which limits their adaptability in real-time implementation with large-scale deployments.
- Dual clustering algorithms are effective in avoiding frequent re-clustering, but they increase communication delay due to complexity in the CH selection process.
- The multi-hop clustering algorithms exist with a hotspot problem, where the energy on the sink nodes drains faster than the usual nodes.
- The use of bio-inspired algorithms is found to optimize the CHs in a better way, but it is not suitable for any generalized applications, as it requires frequent adjustments on multiple tuning parameters.
- The machine learning-based approaches seem to be faster and more active than the traditional methods, but its preparation of training data incurs additional overhead, and that makes it unsuitable for real-world dynamic situations.
- The hybrid metaheuristics demonstrate a superior throughput, but they raise the implementation cost and other general factors in the network scenario.

## 3. Methodology

The DAAC+VC algorithm systematically addresses the research issues of the existing approaches by incorporating residual energy as a percentage of the initial node energy of 0.5 J, node density, and link quality from the network area. This ensures the network region has a perfectly positioned node as a CH with enough residual energy for increasing the network lifetime to certain extent. Moreover, the proposed model prevents the premature CH deaths and cluster imbalance problem by employing a Voronoi-based clustering with a size-limiting constraint. This reduces the overload possibility in certain cluster regions by dividing the nodes more evenly than the existing approaches. Moreover, the proposed approach is designed to ensure a reliable cluster membership under sparse conditions, by making the CHs that fail due to insufficient energy, into a combination with the nearest CH assignment. To address the link distance transmission and hotspot issues, the work utilizes a multi-hop CH for base station communication, which communicates through relay selection among the neighbour CHs. This prevents the model from developing energy holes near the sink, and that improves the network lifetime. The work is also included with a fixed interval of re-clustering with a threshold value to ensure robustness, with respect to the decrease in node density. Furthermore, to improve the packet delivery reliability, a link quality-aware assignment that ensures a probabilistic transmission under varying distance and interference is included in the work. The developed protocol avoids excessive overhead and ensures scalability, by making the maximum percentage of CHs per round with minimum spacing among the sensor nodes. The combination of these concepts ensures the DAAC+VC achieves a prolonged stability with higher throughput and a better packer delivery ratio over the existing methodologies. To ensure the betterment of the proposed Voronoi cells model, the work is also compared with the regular DAAC that is structured with a density-aware adaptive clustering method. In the same way, the proposed work is also compared with the traditional LEACH and HEED approaches. The following subsections explore the theoretical description of the proposed and verified approaches.

### 3.1. Proposed DAAC+VC

In the proposed work, an optimized clustering protocol is suggested by combining density-aware adaptive clustering (DAAC) with Voronoi cell (VC) models to enhance energy efficiency along with load balancing and reliability in the WSNs.

#### 3.1.1. DAAC

The DAAC protocol is an enhancement of the traditional LEACH and HEED schemes. Here, the energy awareness, node density, and link quality are considered for the cluster head selection process by maintaining the multi-hop inter-cluster communication for distant CHs. This hybrid decision scheme enhances the proposed work to achieve a better packet delivery ratio and network lifetime. Unlike LEACH and HEED, the cluster head selection is performed here, based on a composite fitness metric that balances energy, density, and link quality. Therefore, the CH election metric M(i) is defined as follows:

(1) $$M\left(i\right)\;=\;\;\omega_{E}\cdot \frac{E_{res}(i)}{E_{init}}+\;\omega_{D}\cdot \;\frac{1}{1+\;\rho \left(i\right)}+\;\omega_{Q}\cdot LQ(i)$$

where ρ(i) is the total node density, and LQ(i) = $\frac{1}{1+d_{BS}(i)}$ is the link quality factor which is inversely proportional to the distance to the BS. $d_{BS}(i)$ represents the Euclidean distance between the $i^{th}$ node and the base station, and $\omega_{E}$, $\omega_{D}$, and $\omega_{Q}$ are the weight coefficients.

Nodes are ranked in descending order of M(i). A node becomes a CH if it satisfies two conditions:

- Its energy is above zero;
- It is at least $d_{min}$ distance away from any already selected CH.

This ensures energy-rich and well-distributed CHs across the network area, by maintaining the maximum fraction number of alive nodes. Here, the re-clustering does not occur at every round, and that is maintained by a re-cluster interval $T_{re}$; this ensures the model operates adaptively and effectively.

#### 3.1.2. VC

**Figure 1:**
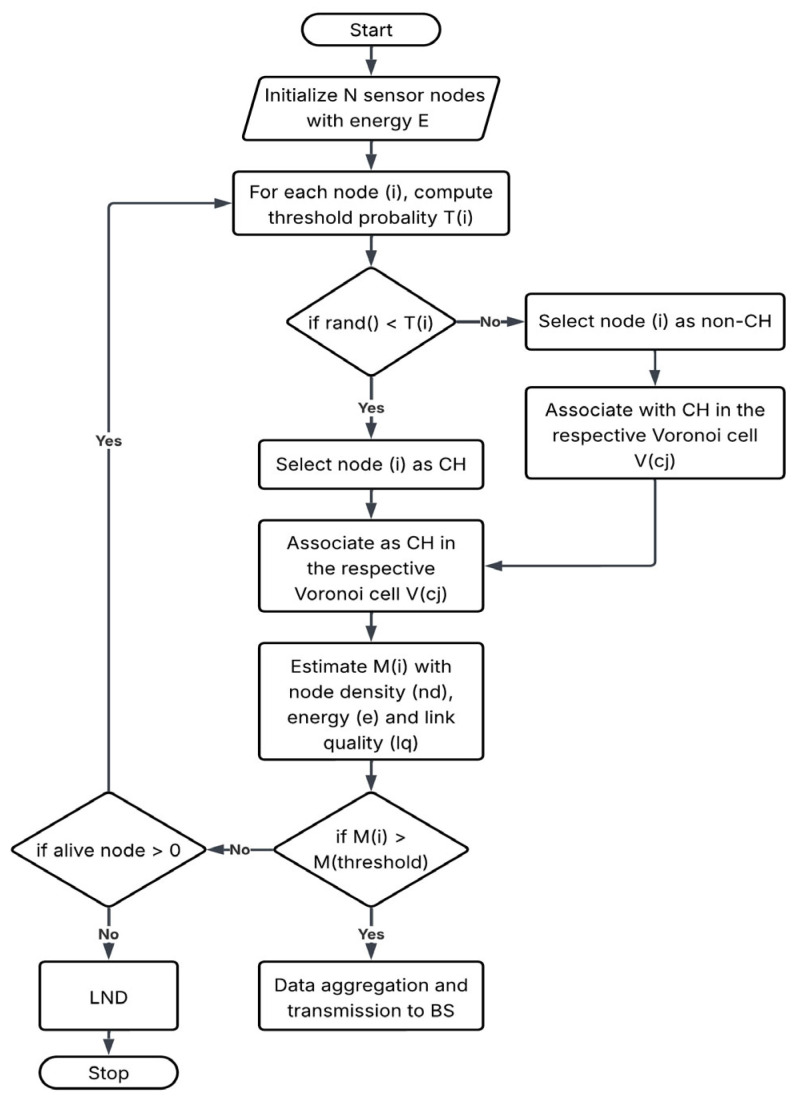
Flowchart of the proposed DAAC+VC.

**Figure 2:**
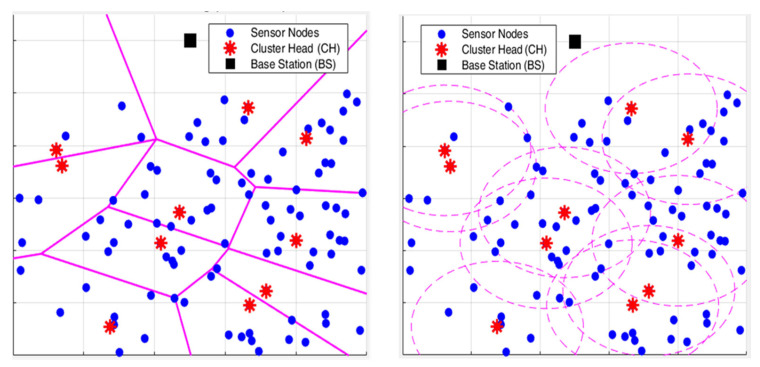
Representation of Voronoi cells mode with the regular clustering in 100 m × 100 m deployment area.

The proposed work considers residual energy, neighbourhood density, and link quality for electing the cluster heads from the network area. It also uses a Voronoi partitioning strategy for maintaining a balanced member assignment. Here, the cluster head selection works the same as in the regular DAAC approach, where N sensor nodes are deployed on a X, Y area with an initial energy of $E_{0}$ along with a base station (BS) located outside the sensing area. The optimal number of cluster heads is not fixed in advance; instead, it is dynamically determined based on the combined influence of residual energy, local node density, and link quality. Nodes with higher residual energy, higher neighbourhood density, and better link quality have a higher probability of becoming CHs. As a result, the number of selected CHs automatically adapts to the network condition and node distribution in each round. Each node is associated with a CH within its communication range, and hence the maximum allowed distance is implicitly limited by the transmission range and linkquality threshold, rather than by a fixed hop count. Figure 1 represents the workflow of the proposed model and Figure 2 indicates a zoom-in view of a network deployed in a 100 m × 100 m network area as mentioned in Table 2. It depicts the clustering model difference between the proposed Voronoi-based DAAC approach and the regular clustering models. The radio model follows the first-order energy model [27], where energy consumed for transmitting the k-bit packet over distance d is as follows:

(2) $$E_{Tx}\left(k,d\right)\;=\;\{\begin{array}{c} kE_{elec}+k\varepsilon_{fs}d^{2},\;d\leq d_{0},\\ kE_{elec}+k\varepsilon_{mp}d^{4},\;d>d_{0}, \end{array}$$

where $E_{elec}$ is the electronics energy, and $\varepsilon_{fs}$ and $\varepsilon_{mp}$ are the amplifier parameters on the free space and multipath models. Therefore, the threshold distance is given by the following:

(3) $$d_{0}\;=\;\;\sqrt{\frac{\varepsilon_{fs}}{\varepsilon_{mp}}}$$

Reception energy is modelled as follows:

(4) $$E_{Rx}\left(k\right)\;=\;kE_{elec}$$

and the cluster head adds additional energy for data aggregation as follows:

(5) $$E_{DA}\left(k\right)\;=\;kE_{DA}$$

After CH selection, clusters are formed based on Voronoi tessellation where the cluster head position C = {c1,c2,…,cm} is determined. Hence, the Voronoi cells of a CH $c_{j}$ is defined as follows:

(6) $$V\;\left(c_{j}\right)\;=\;\;\left\{p\;\epsilon \;R^{2}:\;\left\|p-c_{j}\right\|\leq \left\|p-c_{k}\right\|,\;k\neq j\right\}$$

Here, each alive sensor node is assigned to a CH of its corresponding Voronoi region. To prevent overloading on certain cluster regions, a size-limiting constraint is applied with respect to the distance from the CH, which is less than a maximum radius of $r_{max}$. The model assigns the node to the nearest cluster head association when Voronoi fails due to insufficient cluster head counts or invalid tessellation. A radio model energy cost of $E_{Tx\;}(k,d)$ is utilized for data transmission from all nodes in the cluster to its head. The CH aggregates the data and transmits it to the base station through multi-hop communication along with the nearby CHs on long-distance transmission. Therefore, the work measures throughput on the number of CH to the base station packet deliveries. To prove the efficiency of the proposed DAAC+VC model, it is compared against the traditional models such as LEACH, HEED, and the original DAAC that is structured without Voronoi cells.

### 3.2. LEACH

The Low-Energy Adaptive Clustering Hierarchy (LEACH) protocol is a hierarchical clustering communication technique designed to enhance network lifetime by maintaining energy efficiency. LEACH consists of two phases, namely, the setup phase and the steady state phase, where, in the setup phase, the cluster formation and cluster head selection are performed, and in the steady state phase, the inter-cluster and intra-cluster communication to the base station takes place. A probabilistic-based cluster head selection takes place here, where each node generates a value between 0 and 1 using a uniform random distribution; and, if it falls below the pre-defined threshold value, it is considered as the cluster head for the upcoming rounds. Hence, the threshold function is defined as follows:

(7) $$T\left(n\right)\;=\;\;\{\begin{array}{c} \frac{P_{CH}}{1-P_{CH}+(\gamma \;mod\left(\frac{1}{P_{CH}}\right))},\;if\;n\in G,\;\\ 0,\;otherwise \end{array}$$

Once the cluster head is selected, the remaining nodes choose the nearest cluster head based on Euclidean distance, where each node associates with its respective CH, to minimize the transmission distance, and mathematically, the cluster formation can be represented as follows:

(8) $$CH\;\left(i\right)\;=\;arg\;\underset{\mathrm{j\epsilon C}}{\min}\left(\sqrt{{\left(x_{i}-x_{j}\right)}^{2}+\;{\left(y_{i}-y_{j}\right)}^{2}}\right)$$

where $\left(x_{i}-x_{j}\right)$ represents the coordinates of node i, and C denotes the set of elected cluster heads. This distance-based cluster formation ensures that the member-to-cluster head communication consumes minimal energy. In the steady state phase, each non-cluster head node transmits its sensed data to the associated cluster heads. Hence, the cluster head aggregates the data from its cluster members before transmitting the compressed packet to the base station.

### 3.3. HEED

The Hybrid Energy-Efficient Distributed (HEED) protocol is a distributed clustering technique developed for enhanced energy efficiency and balanced nodes among the sensor nodes, for providing an extended network lifetime in the WSNs. Here, both residual energy and communication cost are considered by the model in cluster head selection and the clustering process. HEED executes iterative rounds in four steps that include initial CH probability estimation, tentative CH selection, final CH confirmation, and cluster formation with data transmission. Therefore, the initial CH probability is estimated based on the following equation:

(9) $$P_{CH}\left(i\right)\;=\;\max\left(C_{prob},\;P_{init}\cdot \frac{E_{res}(i)}{E_{init}}\right)$$

where $P_{init}$ is the initial CH probability, $E_{res}(i)$ is the residual energy of node *i*, $E_{init}$ is the initial node energy, and $C_{prob}$ is the minimum CH probability. Each node probabilistically elects itself as a tentative CH based on $P_{CH}\left(i\right)$, where multiple tentative CHs may emerge within the same vicinity, that may compete at the nearby nodes for the CH role.

## 4. Experimental Analysis

### 4.1. Network Setup

The performance of the proposed DAAC+VC protocol was simulated in MATLAB 2023a in a controlled and uniform network environment for providing an unbiased comparison over the clustering protocols like LEACH, HEED, and DAAC. The proposed DAAC+VC introduces Voronoi-based clustering optimization combined with the regular DAAC that has adaptive cluster formation. Hence, all four of these models were analyzed in an identical initial condition, as stated in Table 2.

Each simulation round consisted of cluster head selection, cluster formation, data aggregation, and intra- and inter-cluster communication. The performance of the network models were analyzed and compared against their number of alive nodes per round, round of first node death (FND), half node death (HND), last node death (LND), average number of cluster heads, packet delivery ratio (PDR), and throughput (packets delivered to BS). These performance analyses provide a holistic assessment of energy efficiency, data reliability, and communication robustness in the WSN.

### 4.2. Results

**Figure 3:**
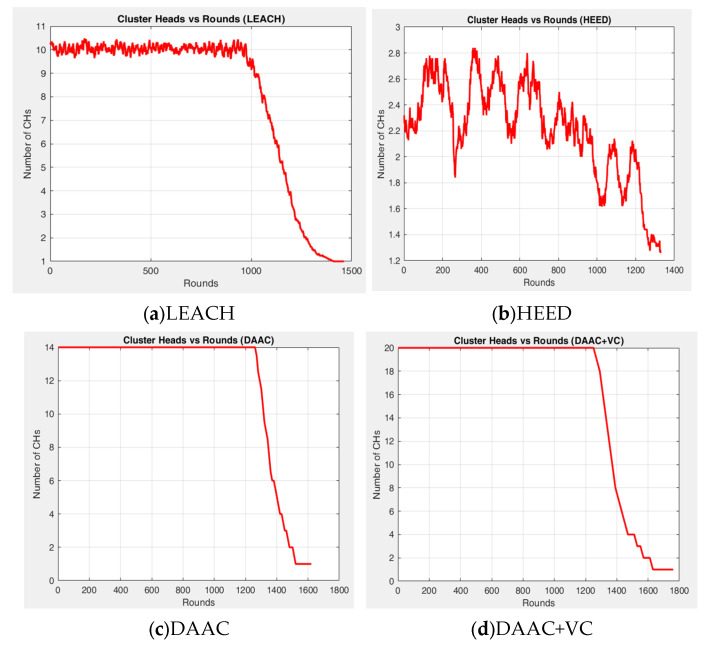
Experimental outcome—cluster heads vs. rounds.

**Figure 4:**
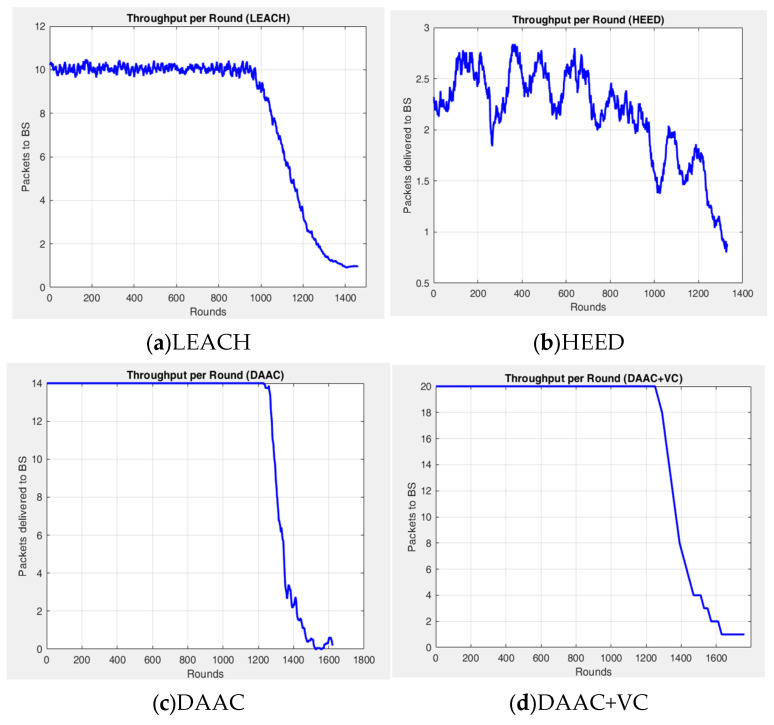
Experimental outcome—throughput vs. round.

**Figure 5:**
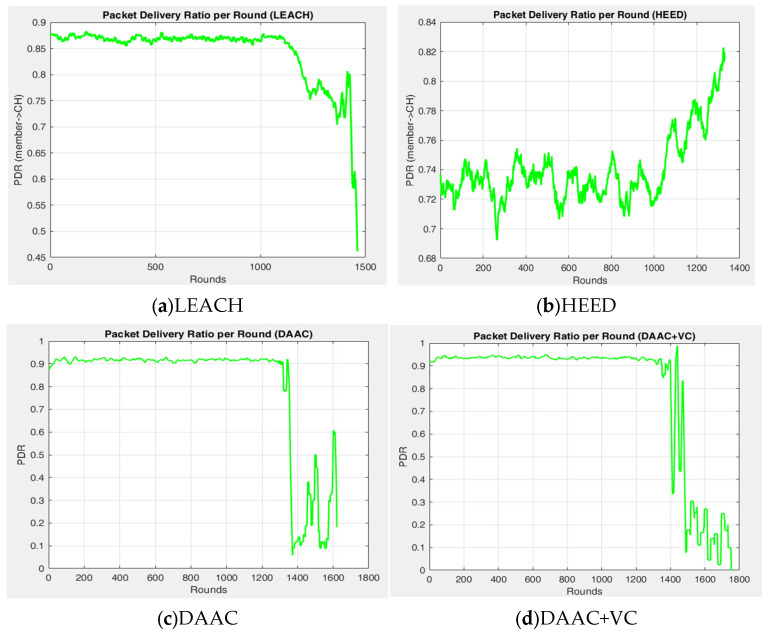
Experimental outcome—PDR vs. round.

Figure 3, Figure 4 and Figure 5 indicate the experimental outcomes of the verified models LEACH, HEED, DAAC, and DAAC+VC on the same network setup. Here, LEACH selects the cluster heads in a probabilistic manner, where each node elects itself as cluster head based on the pre-defined threshold value. Hence, this randomness makes the network to become fluctuated on the cluster head count estimations on each round. In HEED, the cluster head count is estimated based on residual energy and communication cost, and therefore a node with a maximum energy count is always elected as cluster head. This makes the network to operate for a prolonged time. DAAC selects the cluster heads based on an adaptive analysis between distance between the nodes and energy awareness. This helps the model to identify an optimum number of cluster heads per round with limited fluctuations or variability when compared to LEACH and HEED. A Voronoi-based clustering approach is employed on the proposed DAAC+VC model, along with the adaptive cluster head selection. Hence, the occasional imbalance on cluster head selection occurring in the DAAC model is controlled. Additionally, in DAAC+VC, it has also been observed that the model reduces redundancy and scarcity in cluster head selection.

The variation in the number of cluster head count in each model can be viewed in Figure 3, and this happens with respect to the strategy and energy engagement policies of each model. Though the number of cluster head count is almost the same in DAAC and LEACH, in DAAC, the steadiness in the cluster head count is comparatively better and it maintains 14 cluster heads up to 1200 rounds, whereas in LEACH it begins to drop before 1000 rounds. Similarly to DAAC, DAAC+VC also maintains its steadiness over 1200 rounds, but its last cluster head count goes up to 1700 rounds, whereas, in DAAC, it stops at around 1600 rounds. Moreover, the cluster head count in DAAC+VC also reaches a higher count of 20 numbers, due to the implementation of the Voronoi-based clustering model.

Figure 4 represents the performances of the verified algorithms on throughput on each round. In all subplots of Figure 4, the *y*-axis denotes the number of packets delivered to the BS and the *y*-axis range is adapted for each protocol to clearly show its throughput pattern. LEACH indicates a strong fluctuation on its throughput performance because of its randomized and distributed clustering approach. This makes the model to continuously rotate the cluster heads to avoid single node failure. During the stage of cluster head estimation or identification, the performance on throughput is very low, and once the cluster head node is established, the throughput performance starts to improve and work around 14 to 16 on each round. However, the average throughput of LEACH remains at 8.01 for its complete lifetime of 1461 rounds to reach an overall throughput packet of 11,710. The primary focus of the HEED approach is to minimize the energy consumption. Therefore, the number of cluster head formation in this model is very limited over all the other approaches. Hence, the model sends only an average of 2.14 packets per round, with a total throughput packet of 2857, which is very low among the other methods. In the case of DAAC, the total node operation remains up to 1200 rounds where it sends a steady throughput of 14 packets per round, with an overall average of 11.5 packets for 1621 rounds. This is attained by means of the high stable operation for the longer time due to static clustering formation without frequent re-clustering. This causes the model to reach a rapid dead state for the network within 400 rounds from the first node death at 1200 rounds. Among all four models, DAAC+VC performed best, and it sends a steady throughput of 20 up to 1251 rounds due to its higher stability than the original DAAC because of the clustering process through the Voronoi model. This geometric approach accommodates the network in different discrete regions, and that provides a better energy consumption, facilitating the model to run for a longer network lifetime of 1761 rounds.

The comparative analysis on PDR is shown in Figure 5 for all four models. Here, LEACH achieved a moderate PDR value between 0.78 and 0.95, and that is found to be reasonably good in the initial rounds, but its performance was reduced with respect to the individual nodes’ energy depletion. The randomized network cluster head selection followed in LEACH makes the model work on an uneven load distribution, resulting in a variable PDR. HEED is the approach that attained a poor PDR between 0.58 and 0.85, and its deterministic cluster head selection based on residual energy seems to improve the network stability on each round, but its increased overhead causes heavy packet loss in the dense network model, and that results negatively in attaining a better PDR. DAAC achieves a better PDR over the LEACH and HEED, and its PDR ranges from 0.82 and 0.95, where it controls packet loss through the adaptive clustering model and energy-aware communication strategies. DAAC+VC attains a better PDR among all four models, ranging from 0.88 and 0.96, through its dynamic load balancing process in the network scenario. In comparison, the overall average PDR of DAAC+VC remains higher with 0.93, as stated in Table 3.

**Figure 6:**
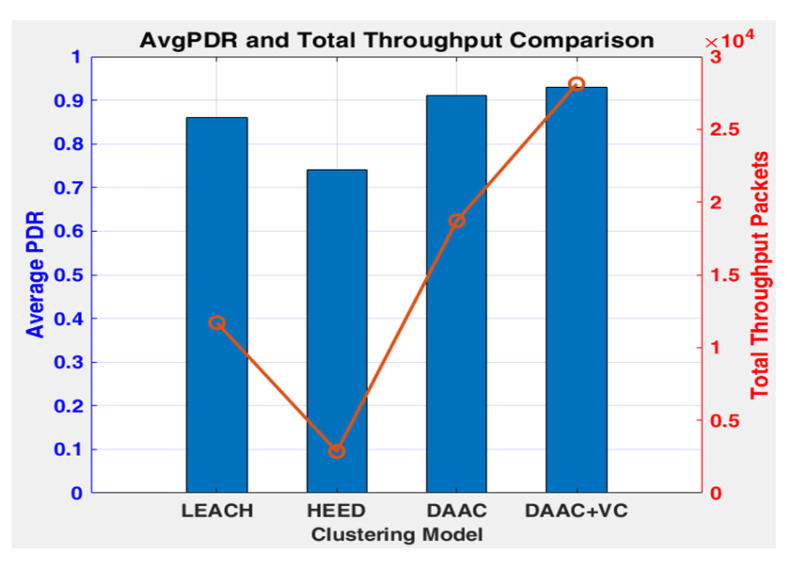
Comparison of PDR vs. throughput.

**Figure 7:**
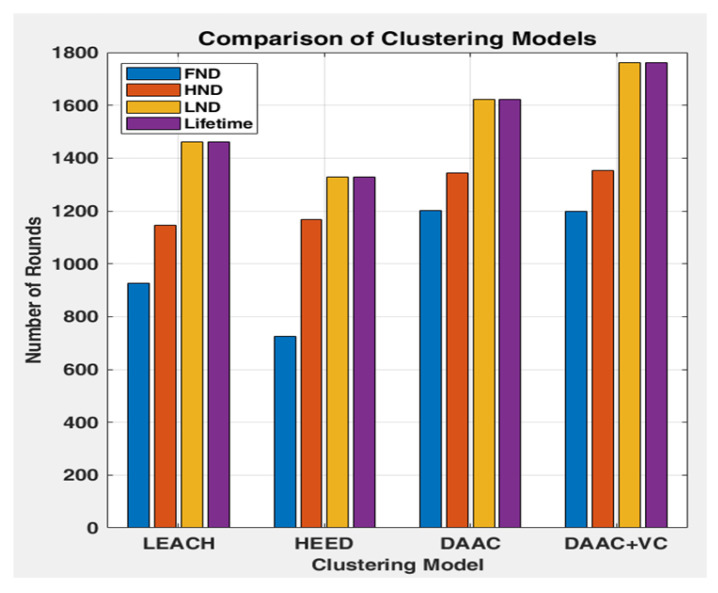
Comparison of network lifetime.

Though the average PDR of DAAC seems to be equal to DAAC+VC, its total throughput packet transmitted count of 18,708 is very much less, and it is 9413 packets less than DAAC+VC, which is graphically presented in Figure 6. Additionally, the network lifetime of DAAC+VC is found to be at a higher level of 1761 rounds, which is better among all the four models. Moreover, the FND and HND of DAAC seems to be better and equal to DAAC+VC, and its LND falls very soon before 140 rounds. A detailed comparison in the network lifetime and node dead at rounds is presented in Figure 7.

## 5. Conclusions

This research introduces DAAC+VC, an enhanced version of the DAAC protocol that utilizes Voronoi cell regions for an optimized clustering process. The simulation results indicate that the performances of DAAC outperform those of the traditional clustering algorithms LEACH and HEED in a remarkable way, with an improved network lifetime of 10.95% and 21.97%, respectively. Furthermore, the performance of the proposed DAAC+VC extends its network lifetime over the regular DAAC with an improvement of 8.64%, along with a better average PDR of 2.2%, and, notably, it improves the total throughput packets by 50.31%. This indicates that the proposed DAAC+VC has a better capability of handling high network traffic in a dense network region. These findings also confirm that the integration of Voronoi-based clustering into the DAAC protocol is a highly effective approach for enhancing the overall performance and longevity of the dense wireless sensor networks. In future, the proposed DAAC+VC framework can be further extended by incorporating more advanced multicriteria decision-making models, such as fuzzy logic-based decision frameworks, to improve the accuracy of cluster head selection under highly dynamic network conditions. In addition, the integration of uncertainty-aware models and probabilistic optimization techniques such as Bayesian learning and stochastic optimization can be explored to handle unpredictable node behaviours and varying link conditions more effectively. The applicability of the proposed approach can also be validated on large-scale real-time deployments and heterogeneous WSN environments to assess its practical scalability and robustness.

## Figures and Tables

**Table 1:** Comparative analysis of existing WSN clustering techniques.

Ref	Technique/Model	Energy Consumption per Round	Network Lifetime Improvement	Throughput	Major Contribution/Limitation
[9]	PSO-based Adaptive Clustering	Reduced	↑ 20% vs. Fuzzy, ↑ 10% vs. K-means PSO	Moderate	Improved network longevity; suffers from premature convergence
[13]	Whale Optimization for CRSN	Reduced	Not explicitly reported	↑ 15%	Optimized energy and cluster size; overhead still exists
[14]	Ink Drop Spread Energy-Aware Clustering	Reduced	↑ 17% vs. LEACH and PEGASIS	Moderate	Better residual energy and active nodes; dynamic clustering overhead
[15]	Improved Squirrel Search Algorithm	Low (210 mJ)	Indirectly improved	Moderate	High PDR (88%); cluster formation still computation-intensive
[16]	Q-learning + Artificial Bee Colony	Very Low (0.253 units)	Improved vs. LEACH and HEED	Moderate	Better routing reliability; training overhead exists
[17]	Intra-Cluster Multi-hop CH Rotation	Reduced	Improved	Moderate	Reduced re-clustering; threshold tuning is critical
[18]	Multi-level Clustering + Pufferfish Optimization	↓ 29%	↑ 62.5%	Moderate	Handles premature convergence; complexity increases
[19]	Fuzzy C-Means + Sailfish–Whale Optimization	↓ 50% vs. WOA	Improved	High	Better PDR and throughput; parameter tuning dependency
[20]	Spotted Hyena Optimization	Reduced	First node failure at 1300 rounds	Moderate	Improved stability; deployment cost still exists
[21]	Transient Search Optimization	Reduced	↑ 56.37%	Moderate	Efficient lifetime extension; limited scalability study
[22]	Mega-Cluster-Based Routing	Reduced	↑ 34.5%	Moderate	Hotspot mitigation; centralized clustering overhead
[23]	Spider Wasp Optimizer-Based Multi-hop Routing	Reduced	↑ 32.7%	Moderate	Improved lifespan; relay dependency exists
[24]	ANN-Integrated LEACH	Reduced	Indirect improvement	Moderate	Fast CH classification (85% accuracy); training overhead
[25]	Dual CH + Hybrid Metaheuristics (Cheetah + FPA + CPA)	Reduced	1270 rounds	Moderate	High residual energy; high implementation complexity
[26]	Multi-objective Deep CNN + Hybrid Optimization	Reduced	↑ 50% alive nodes	High	Superior performance; very high computational overhead

↑ represents increase and ↓ represents decrease.

**Table 2:** Network Setup.

Parameter	Value
Number of sensor nodes	200
Deployment area	100 m × 100 m
Initial energy per node	0.5 J
Data packet size	4000 bits
Energy consumed in transmission (ETX)	50 nJ/bit
Energy consumed in reception (ERX)	50 nJ/bit
Data aggregation energy (EDA)	5 nJ/bit/signal
Free space amplifier (εfs)	10 pJ/bit/m^2^
Multipath amplifier (εmp)	0.0013 pJ/bit/m^4^
Simulation rounds	Until all nodes are dead (lifetime)

**Table 3:** Performance comparison of the verified models.

Clustering Model	FND	HND	LND	Lifetime	Avg. PDR	Total Throughput Packets
**LEACH**	927	1146	1461	1461	0.86	11,710
**HEED**	725	1168	1329	1329	0.74	2857
**DAAC**	1203	1344	1621	1621	0.91	18,708
**DAAC+VC**	1198	1353	1761	1761	0.93	28,121

## Data Availability

The original contributions presented in this study are included in the article. Further inquiries can be directed to the corresponding author.
